# Who Is Worst Off? Developing a Severity-scoring Model of Complex Emergency Affected Countries in Order to Ensure Needs Based Funding

**DOI:** 10.1371/currents.dis.8e7fb95c7df19c5a9ba56584d6aa2c59

**Published:** 2015-11-03

**Authors:** Anneli Eriksson, Ylva Kristina Ohlsén, Richard Garfield, Johan von Schreeb

**Affiliations:** Public Health Science, Centre for Research on HealthCare in Disasters, Health Systems and Policy Research Group, Karolinska Institutet, Stockholm, Sweden; Technology and Health, Red Cross University College, Stockholm, Sweden; ERRB, Centers for Disease Control, Atlanta, Georgia, USA; Department of Public Health Sciences, Health System and Policy, Karolinska Institute, Stockholm, Sweden

## Abstract

Background: Disasters affect close to 400 million people each year. Complex Emergencies (CE) are a category of disaster that affects nearly half of the 400 million and often last for several years. To support the people affected by CE, humanitarian assistance is provided with the aim of saving lives and alleviating suffering. It is widely agreed that funding for this assistance should be needs-based. However, to date, there is no model or set of indicators that quantify and compare needs from one CE to another. In an effort to support needs-based and transparent funding of humanitarian assistance, the aim of this study is to develop a model that distinguishes between levels of severity among countries affected by CE.

Methods: In this study, severity serves as a predictor for level of need. The study focuses on two components of severity: vulnerability and exposure. In a literature and Internet search we identified indicators that characterize vulnerability and exposure to CE. Among the more than 100 indicators identified, a core set of six was selected in an expert ratings exercise. Selection was made based on indicator availability and their ability to characterize preexisting or underlying vulnerabilities (four indicators) or to quantify exposure to a CE (two indicators). CE from 50 countries were then scored using a 3-tiered score (Low-Moderate, High, Critical).

Results: The developed model builds on the logic of the Utstein template. It scores severity based on the readily available value of four vulnerability and four exposure indicators. These are 1) GNI per capita, PPP, 2) Under-five mortality rate, per 1 000 live births, 3) Adult literacy rate, % of people ages 15 and above, 4) Underweight, % of population under 5 years, and 5) number of persons and proportion of population affected, and 6) number of uprooted persons and proportion of population uprooted.

Conclusion: The model can be used to derive support for transparent, needs-based funding of humanitarian assistance. Further research is needed to determine its validity, the robustness of indicators and to what extent levels of scoring relate to CE outcome.

## Background

A disaster is as an event that overwhelms local capacity, necessitating national or international assistance 1. According to CRED, an estimated 400 million people annually are affected by disasters, of which over 170 million are affected by conflicts 2 ^,^ 3 ^,^ 4. The Utstein template categorizes disasters based on the type of risk and hazard causing them 5. There are natural, manmade and mixed disasters. Complex emergencies (CE) are a manmade disaster, defined as a situation where civilian mortality due to direct or indirect causes of conflict has increased significantly 6. Disasters overwhelm existing capacities and require relief in the form of humanitarian assistance. The objective of humanitarian assistance is to save lives, alleviate suffering, and maintain human dignity 7. In 2013, at least 22 billion USD worth of humanitarian assistance was globally made available to assist disaster affected populations 8. Two thirds of this sum was from governmental donors and mainly allocated to CE 8. Donors have long agreed that funding for humanitarian assistance should be allocated according to, and in proportion to, needs 7 ^,^ 9.

However, there is no commonly accepted definition of “need”. Maslow’s pyramid categorizes human needs in a hierarchy, where the physical needs to achieve survival are the base, followed by safety, social needs, esteem and self-actualization at the top 10. In development and humanitarian aid, the concept of *basic needs* has been developed, mainly referring to basic services required for a community, including food, shelter, and clothing for the individual. A donor often receives plentiful information on these aspects of need in requests for funding 9 ^,^ 11. Still, studies have documented the lack of a systematic approach to defining the relative importance and proportion of needs between disasters as a basis for funding 12 ^,^ 13. A main challenge for needs-based funding is the lack of commonly accepted indicators that define and quantify needs. A recent study highlighted the urgent need for defined, specific and well-accepted indicators and a system to determine the severity of crises and allow comparisons between disaster-affected countries 13.

Existing frameworks, such as ECHO’s Global Needs Assessment (GNA), the Inform index for risk management, and ACAPs Global Emergency Overview (GEO), typically rate CE countries in the "worst off" category, underlining the need for humanitarian assistance in these countries 14 ^,^ 15 ^,^ 16. However, while these initiatives allow for a broad comparison between the worst off countries, they do not distinguish between the levels, severity, and magnitude of need among the countries. UNOCHA’s Global Focus Model (GFM) focuses on risk, but is not specifically designed to inform about the intensity or severity of an on-going CE 17. Bayram et al. propose an assessment tool for CE, based on scoring of a limited number of indicators, but the tool is based on data from sudden onset disasters, not CE, and several of the indicators are not readily available. Thus the tool has limited value in supporting needs-based funding decisions 18.

Therefore, the basis of this study is the apparent need for a practical tool to assess severity and levels of need in CE affected countries. The aim is to develop a severity scoring model, built on well-defined and readily available indicators, that can facilitate decision making for needs-based allocation of humanitarian assistance funding.

## Materials and Methods

***Study assumptions and preconditions***

Severity is a predictor of level of need. The level of severity is dependant on vulnerability and exposure 5. A limited number of recognized and readily available indicators can be used to characterize and quantify vulnerability and exposure. Textbox 1 describes the Utstein template and defines the components and other terms that capture disaster severity.

**Figure:**
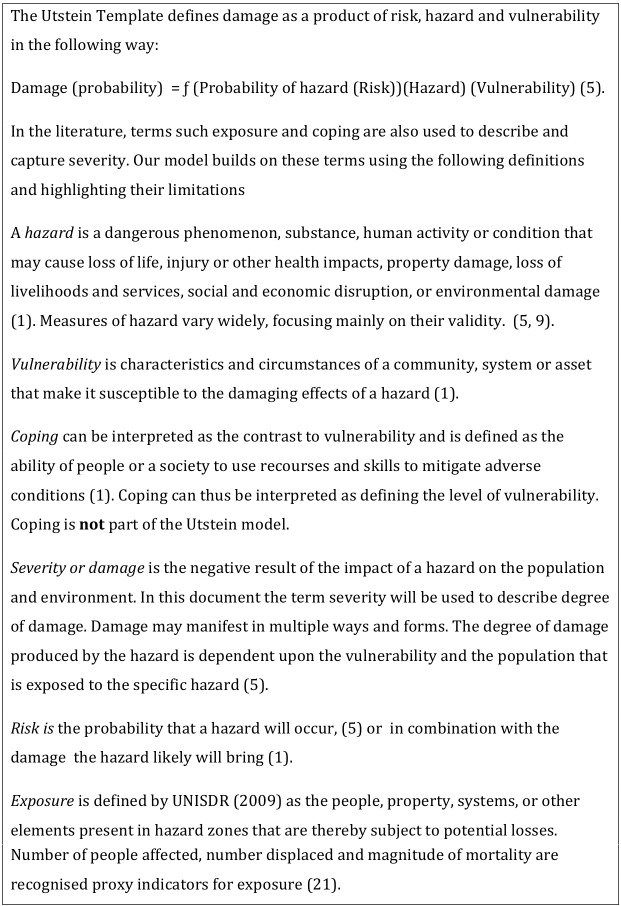
**Textbox 1. Model and concepts**

A practical scoring model was defined as one that a) can be populated with data within a few hours, b) uses indicators that are readily available, and c) provides a numerical result.

**Figure:**
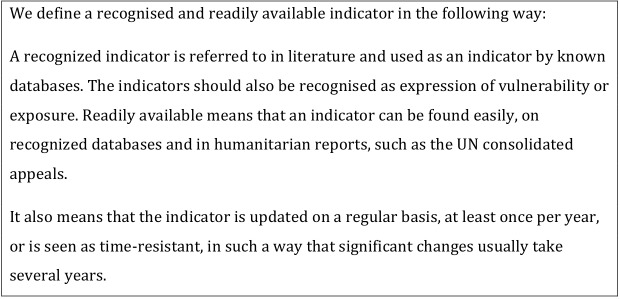
**Textbox 2. Recognised and readily available indicators**

**Process**

The following process was laid out: 1) a two-step Internet and document search, 2) the extraction, listing and ranking of indicators, 3) outlining a preliminary model based on the Utstein template logic, 4) modification in line with the model's precondition of easy to use, and population of the final model with the selected indicators, and 5) definition of cut-off levels for the indicators.

1) To generate a list of well defined, commonly used vulnerability indicators that were defined and assessed in the scientific literature, an Internet search was done using the EBSCO database, which includes preMEDLINE and MEDLINE and Google scholar, without any time limit. Searches were limited to English using the search words: Disaster(s), Emergency(ies), Vulnerability, Index, and Indicator(s) in different combinations.

To assess the use and availability of vulnerability and severity indicators in Consolidated Appeals (CAP) (United Nations proposals for humanitarian funding), documents from 2010 and 2012 19 ^,^ 20 were searched, using the same criteria as in the Internet search.

The first step of the Internet and document search was to identify vulnerability indicators that fit the search criteria and the purpose of the study. In the second step, 27 CAP documents were searched, and shortlist was made of the indicators that were used in more than one third of the documents.

2) The indicators from the two-step search were compiled and ranked according to: relevance and relation to best practices or evidence; timeliness; and availability.

The indicators were ranked from 1 – 3 for each area above, which gave a possible score range of 3 to 9 points for each indicator. To calculate the rank for best available practice, three points were given to indicators that were suggested in at least three individual articles from the literature search and used in at least three of the searched indexes (see table 1). Two points were scored to indicators suggested in at least two articles and used in two indexes while one point was given to indicators that were suggested in less than two articles. To calculate the rank for timeliness, three points were given to indicators likely to be updated when a context changes, or updated more frequently than annually. Two points were allocated to indicators that normally are updated annually and one point for less frequent updates. Ranking of availability or likelihood of availability was done by scoring three point for indicators found in more than one third of the CAP appeals searched and indicator and in World Bank statistics data base, two points if the indicator was found in one of two above and one point if the indicators were unavailable in both.

Vulnerability and exposure indicators were selected based on ranking. In our selection we also aimed for a variation between different fields (i.e. economics, health, education, and so on) rather than selecting indicators from the same field even if they ranked highly.

3) A preliminary model based on the Utstein template logic was outlined and populated with the indicators selected in step 2 (Textbox 1).

4) To adjust the model to its practical purpose, it was tested and modified so that it should be possible to assess and compare severity within a few hours, use readily available indicators, and provide a numerical result. The final model had two categories: vulnerability and exposure. Based on their relative importance, four indicators for vulnerability and four for exposure were selected.

5) Distinguishing levels of severity

To allow comparison and to distinguish the level of severity between CE countries, we set a three-tiered scoring system for the value of each indicator: Low-Moderate, High, and Critical. The scoring values of the vulnerability indicators were built on values from approximately 50 countries with a low development index (a value of less than 0,5 by the UNDP), while exposure values used data from the 15 CAPs for 2012.

## Results

Part one of the results section shows the process leading up to the selection of indicators. Part two shows the completed model.

**Part one**

The results of the two-step Internet and document search are presented in Tables 1 and 2. A total of 19 single indicators were identified as valid in relation to vulnerability; 17 of them were used in vulnerability and development indexes. In addition, 3 of them also primarily indicated exposure. We found 14 indicators and one index (HDI) that were used for at least 9 countries in the 2010 and 2012 UN CAP documents. The tables display the indicators per sector. Eight indicators were the same or similar in the two searches. These are highlighted in bold, in Table 2.

**Figure:**
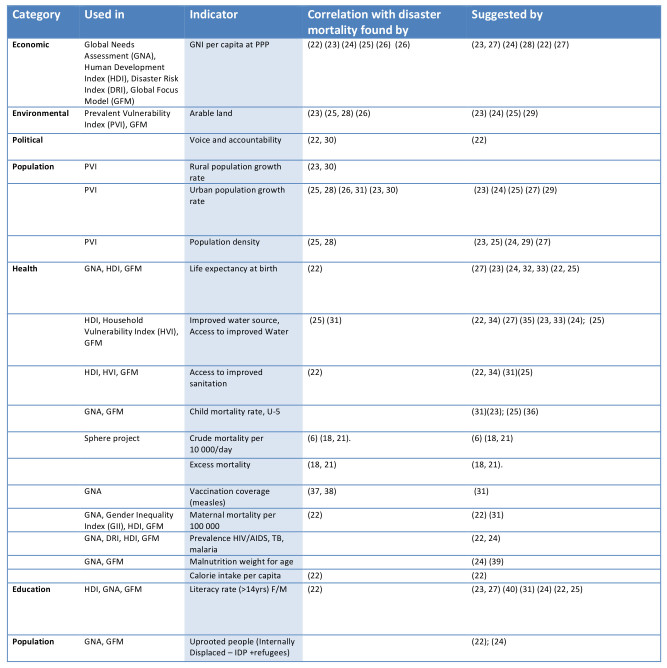
**Table 1. Seventeen single indicators identified as valid in relation to vulnerability.**

**Figure:**
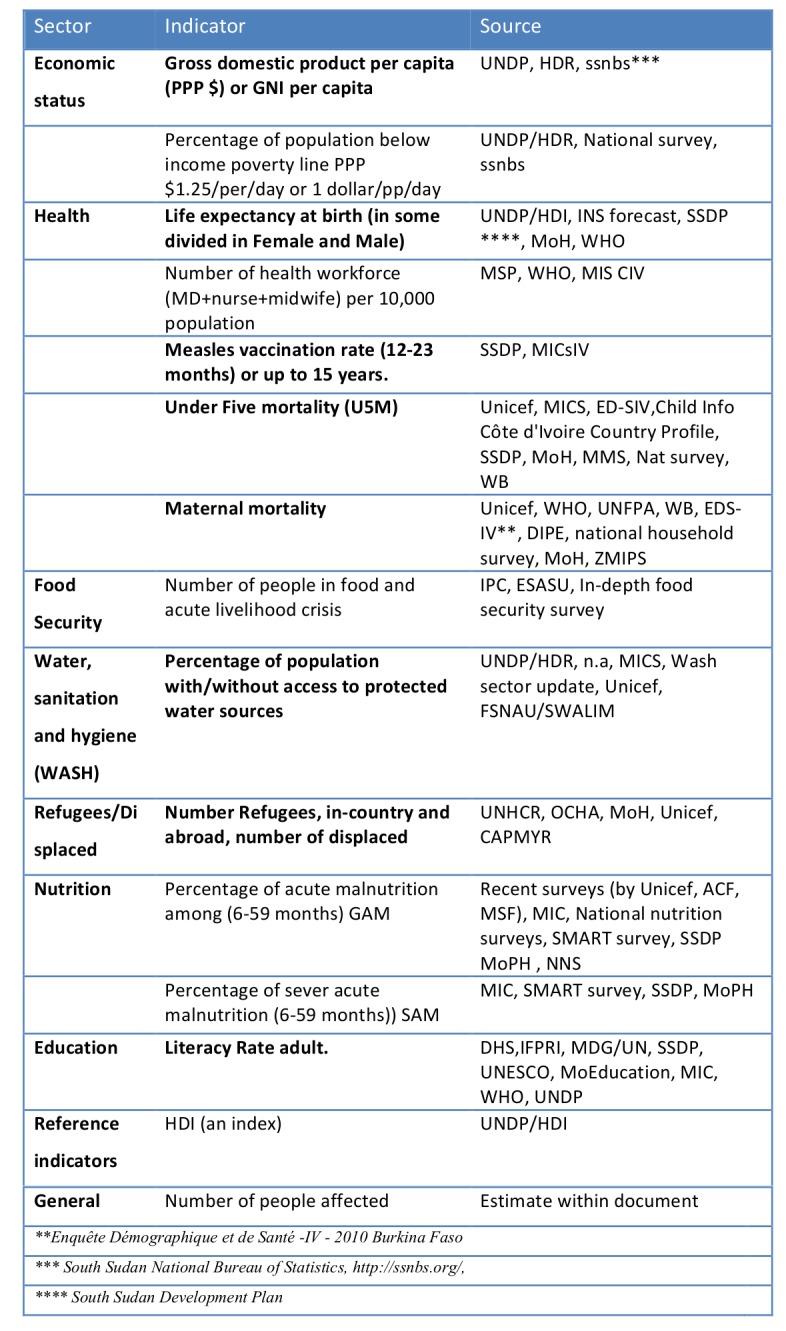
**Table 2. Indicators used to capture the situation for at least nine countries of the 2010 and 2012 UN Consolidated Appeal documents.**

Table 3 ranks the 25 indicators. While some ranked high as relevant in relation to best practices or recognized evidence, the timeliness and availability were rated low. For instance, this was the case for excess mortality and crude mortality rates per 10 000 people per day.

**Figure:**
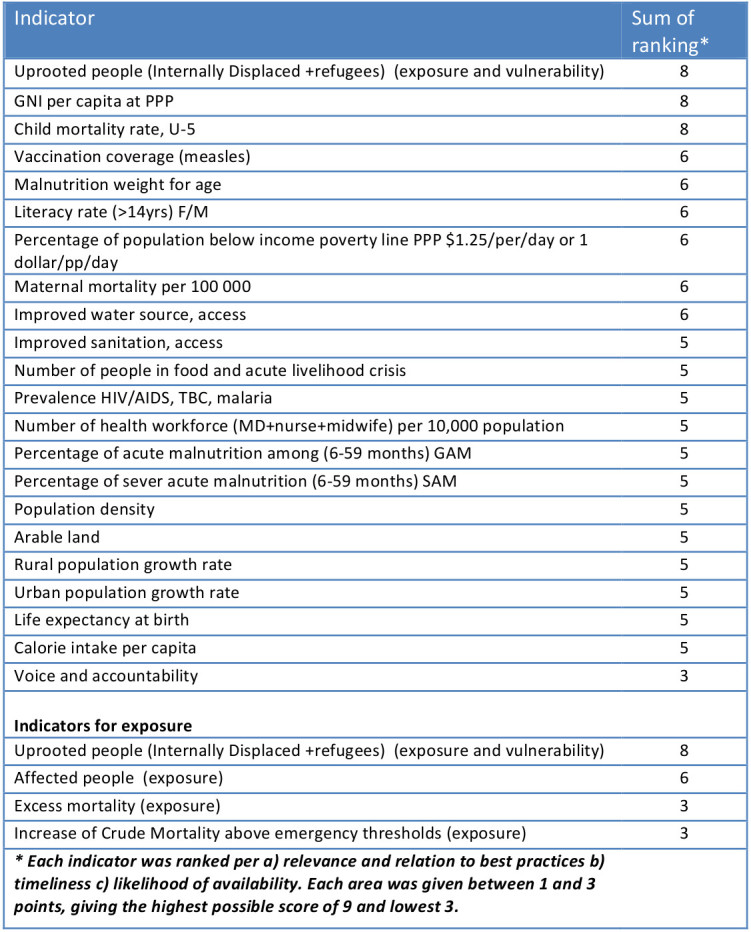
**Table 3. Ranking of indicators for vulnerability and exposure**

**Part two**

The final model consists of the formula Disaster severity = f (Vulnerability) (Exposure)

**Figure:**
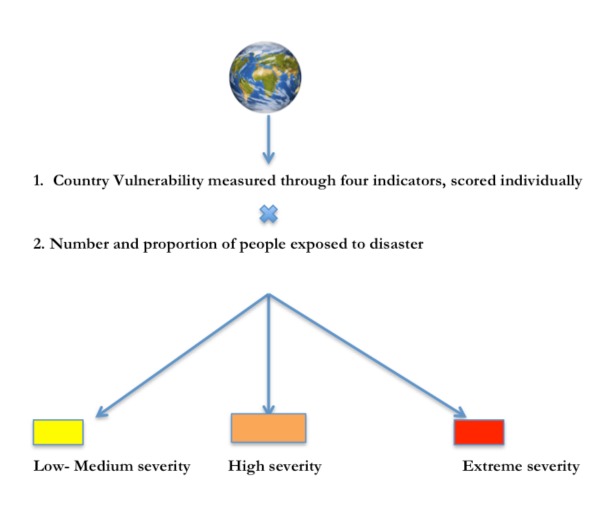
**Fig. 1: Illustration of the model**

This is a simplification of the Utstein model. For a detailed description of how the model was simplified, see ANNEX I.

Of the top ranked indicators in Table 3, six were selected for the model. The selected vulnerability indicators were: 1) GNI per capita, 2) PPP Under-five mortality rate, per 1000 live births, 3) adult literacy rate, % of people ages 15 and above, and 4) underweight, % of population under 5 years.

The selected exposure indicators were: 5) total number of affected people, also as proportion of the total population, and 6) total number of uprooted people, also as proportion of the total population.

These indicators met the inclusion criteria and provide information on differing aspects of vulnerability and severity. ANNEX II has definitions of the indicators. Tables 4 and 5 illustrate the the scoring of the indicators. It should be noted that scoring Low-Medium does not indicate an acceptable situation, but that the country scored less poorly than those scoring High or Critical.

**Figure:**
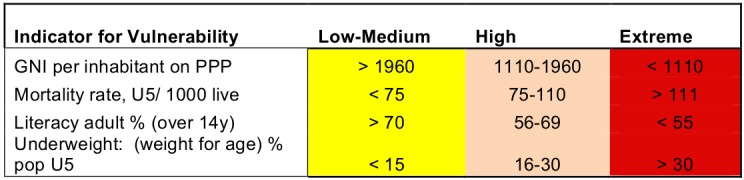
**Table 4. Scoring of the vulnerability indicators**

**Figure:**
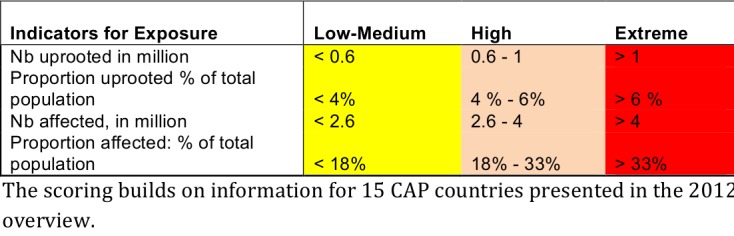
**Table 5. Scoring of indicators for exposure**

Finally, the model was populated with the proposed indicators an suggested scoring levels.

**Figure:**
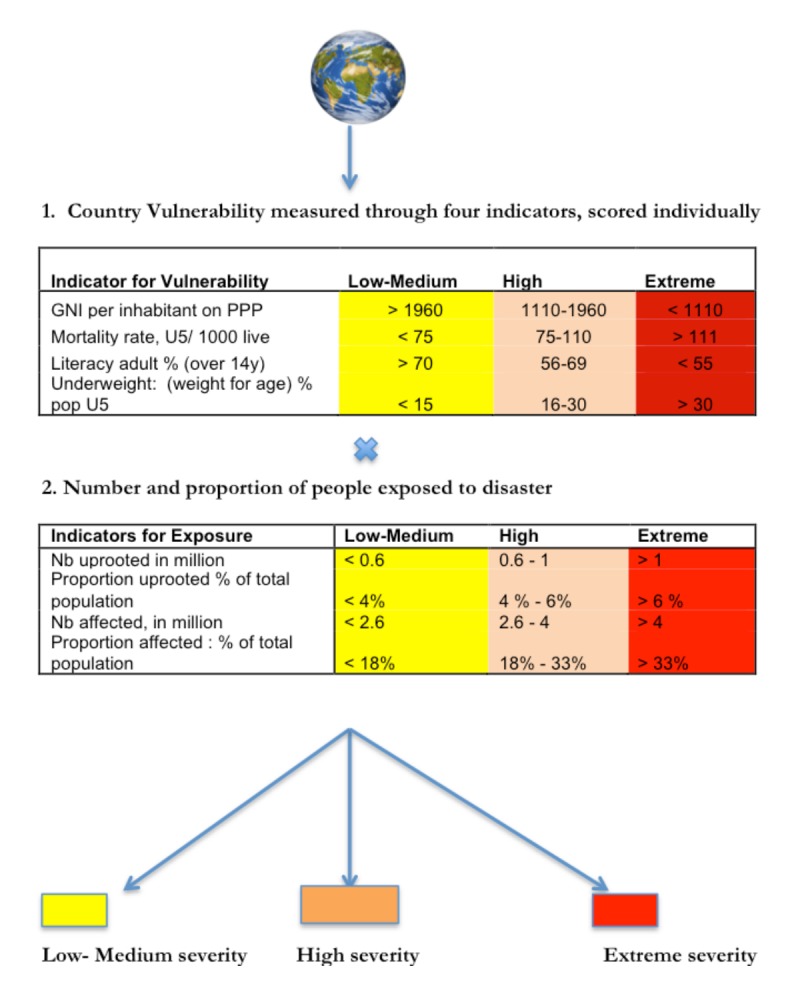
**Fig. 2: Illustration of the model with indicators and scoring**

## Discussion

To our knowledge, this is the first model that compares severity and needs between complex emergency affected countries in a systematic and transparent way. It provides an objective tool, based on accepted and available indicators, that can support needs-based funding decisions for humanitarian assistance. However, it should be noted that this type of support tool, based on quantitative indicators, cannot replace the analytical work being done by competent staff at humanitarian funding agencies. We are aware of the ethical challenge of putting numbers on human suffering and scoring the severity of already poor and conflict-affected countries. Nevertheless, a needs-based funding policy must be accompanied by a transparent account of the basis on which funding decisions are made.

This study has several limitations. It is based on the assumption that needs and level of severity are interchangeable. A small group of experts, who were also the authors, selected the indicators and developed the model. However, the experts, who all have experience with CE, needs assessments, and academia, have explained in detail the process of selection for the indicators and priorities.

The main challenge for this model has been how to balance pragmatism with robust scientific methods. It was important to develop a model that could be used easily, rather than a complex model that may have strong theoretical validity, but lack practical feasibility. The challenge was to get a balance between what we would have liked to have and what is feasible to get. We have carefully developed a model that balances optimal ignorance and appropriate imprecision with the increasing opportunistic costs of being more precise. Another limitation is that the model assumes that the values of the indicator are based on reliable data. Nevertheless, the indicators are the “best available” and are what pledges and allocations are built on. We also removed aspects of the original Utstein template that did not have a reliable numeric indicator. These non-quantifiable aspects should be included in the qualitative analytical work done by donors before they decide on what country is more in need. Even with a general consensus that funding should be needs-based, we assume that political aspects are taken into consideration when funding decisions are made. Our model does not cover these aspects, but could be used as a objective and transparent tool to balance this.

The suggested model will need testing and validation to show its value. In forthcoming work, we explore the relevance of the model in defining severity and needs by applying it to ongoing CE, as well as on historical CE data. We welcome colleagues to comment and provide input to improve the model. We are convinced that a solid and accepted model that enables donors to direct funding to those most in need has the potential to improve humanitarian assistance, save lives and alleviate suffering.

## ANNEX 1 Development of Conceptual models for severity scoring of complex emergencies

ANNEX 1

## ANNEX 2 Indicators, their meaning and relevance

ANNEX 2

## Competing Interests

The authors have declared that no competing interests exists.
